# Rooting the eutherian tree: the power and pitfalls of phylogenomics

**DOI:** 10.1186/gb-2007-8-9-r199

**Published:** 2007-09-21

**Authors:** Hidenori Nishihara, Norihiro Okada, Masami Hasegawa

**Affiliations:** 1 Graduate School of Bioscience and Biotechnology, Tokyo Institute of Technology, 4259-B-21 Nagatsuta-cho, Midori-ku, Yokohama 226-8501, Japan; 2 Department of Statistical Modeling, Institute of Statistical Mathematics, 4-6-7 Minami-Azabu, Minato-ku, Tokyo 106-8569, Japan; 3 School of Life Sciences, Fudan University, Handan Road 220#, Shanghai 200433, China

## Abstract

**Background:**

Ongoing genome sequencing projects have led to a phylogenetic approach based on genome-scale data (phylogenomics), which is beginning to shed light on longstanding unresolved phylogenetic issues. The use of large datasets in phylogenomic analysis results in a global increase in resolution due to a decrease in sampling error. However, a fully resolved tree can still be wrong if the phylogenetic inference is biased.

**Results:**

Here, in an attempt to root the eutherian tree using genome-scale data with the maximum likelihood method, we demonstrate a case in which a concatenate analysis strongly supports a putatively wrong tree, whereas the total evaluation of separate analyses of different genes grossly reduced the bias of the phylogenetic inference. A conventional method of concatenate analysis of nucleotide sequences from our dataset, which includes a more than 1 megabase alignment of 2,789 nuclear genes, suggests a misled monophyly of Afrotheria (for example, elephant) and Xenarthra (for example, armadillo) with 100% bootstrap probability. However, this tree is not supported by our 'separate method', which takes into account the different tempos and modes of evolution among genes, and instead the basal Afrotheria tree is favored.

**Conclusion:**

Our analysis demonstrates that in cases in which there is great variation in evolutionary features among different genes, the separate model, rather than the concatenate model, should be used for phylogenetic inference, especially in genome-scale data.

## Abstract 2

In an attempt to root the eutherian tree using genome-scale data with the maximum likelihood method, a concatenate analysis supports a putatively wrong tree, whereas separate analyses of different genes reduced the bias.

## Background

In the post-genomic era, genome-scale approaches to phylogenetic inference (phylogenomics) are being applied extensively to overcome the large sampling errors inherent in commonly used approaches based on a single or a small number of genes [1-3]. Sampling error diminishes as the number of genes provided for the analysis increases, but the fully resolved tree can still be wrong if the phylogenetic inference is biased (systematic error), and several such cases have been reported [4-11]. To estimate a reliable tree from large genomic datasets, it is imperative to establish how best to overcome such an error. Currently, genome projects of various mammalian species are ongoing at a rapid pace, and their genome-scale sequence data are now available. Therefore, an analysis of mammalian phylogeny based on such datasets is expected to be useful in evaluating problems that are inherent to phylogenomics.

Mammalian phylogenetics has developed rapidly during the past decade, and most of the higher order relationships have been resolved [12-16]. All eutherian (placental) mammals can be classified into 18 orders, which are grouped into the three higher groups: Afrotheria (for example, elephants, sirenians, hyraxes, and so on, which originated in Africa), Xenarthra (for example, armadillos, sloths, and anteaters, which originated in South America), and Boreotheria (all other eutherians, comprising 11 orders that originated in Laurasia of the Northern hemisphere). Phylogenetic relationships have been analyzed primarily using sequences of several nuclear or mitochondrial genes. However, the root of the eutherian tree remains unclear. Even extensive phylogenetic analyses based on several gene sequences failed to resolve the relationship among the three groups [17-21]. On the other hand, two retrotransposon inserted loci analyses have supported the basal Xenarthra hypothesis [15], whereas Murphy and coworkers [22] identified two loci that support the monophyly of Xenarthra and Afrotheria. However, the small number of loci does not provide conclusive evidence to resolve the relationship because of a possible ascertainment bias. The monophyly of Xenarthra + Afrotheria might be considered a reasonable hypothesis from a biogeographic point of view [17], because the South American and African continents - where Xenarthra and Afrotheria, respectively, originated - constituted the supercontinent Gondwana until about 105 million years ago [23]. Indeed, the early split of eutherians is estimated to be about 100 million years ago [24], which is consistent with the biogeographic viewpoint. Thus, rooting the eutherian tree is important not only to clarify the origin of eutherians but also to elucidate the correlation between long-term continental drift and mammalian migration and diversification.

Although genome-scale approaches have become popular during the past few years, at most only a few hundreds of genes (a few hundred kilobases for each species) have thus far been used for phylogenetic inference [1,3,4,8]. In the present study we collected 2,789 genes from ten mammalian genomic sequences by screening whole-genome data, providing 1 megabase (Mb) of sequence data for each species, and performed an extensive maximum likelihood (ML) analysis to determine the root of the eutherian tree.

## Results and discussion

### Megabase data collection to analyze the root of eutherian tree

**Figure 1:**
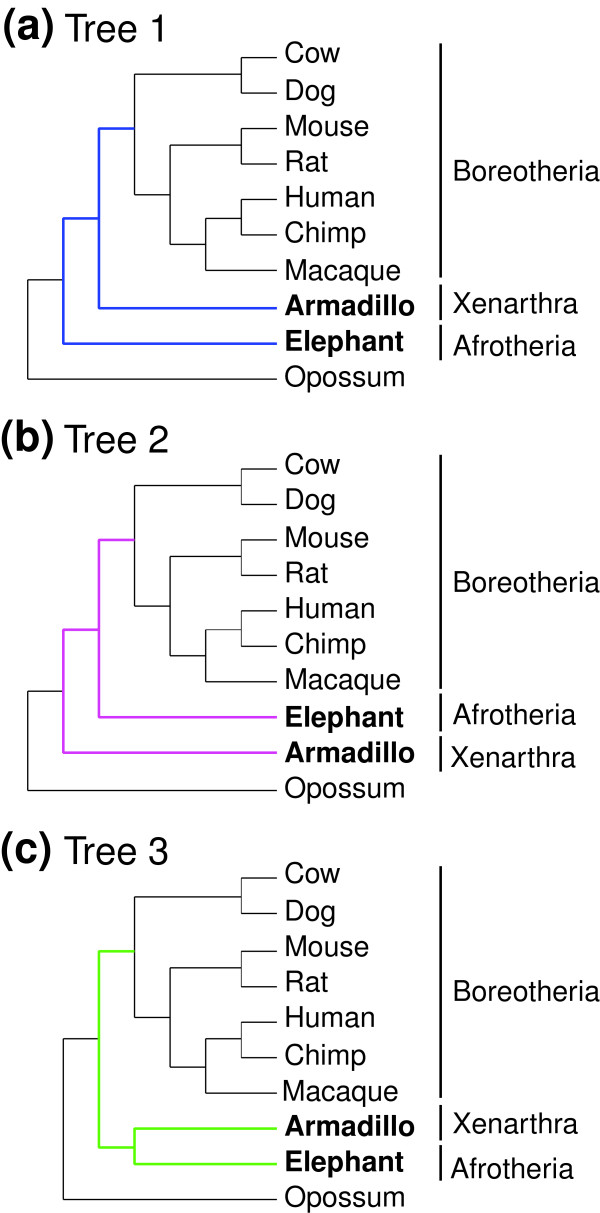
Three phylogenetic hypotheses for the root of theeutherian tree. **(a) **Tree 1: basal Afrotheria. **(b) **Tree 2: basal Xenarthra. **(c) **Tree 3: basal Boreotheria, or Afrotheria/Xenarthra clade. The phylogenetic relationships within Boreotheria (cow, dog, mouse, rat, human, chimpanzee, and macaque) are fixed in this study.

Whole-genome shotgun data from several mammalian species are now available. In this study, we used about 2 gigabases of sequence data for each of the nine-banded armadillo (*Dasypus novemcinctus*) in Xenarthra and the African elephant (*Loxodonta africana*) in Afrotheria. We obtained the armadillo and elephant homologs to the human exons. Subsequently, we extracted the relevant orthologs from a whole-genome alignment of human with chimpanzee, rhesus macaque, mouse, rat, dog, cow, or opossum, and finally we constructed a 1,011,870 base pair (bp; 337,290 amino acids) sequence dataset containing 2,789 genes for each species. In our analysis, three possible trees among Afrotheria, Xenarthra, and Boreotheria were examined: tree 1 was basal Afrotheria, tree 2 was basal Xenarthra, and tree 3 was basal Boreotheria, or Afrotheria/Xenarthra clade (Figure 1). The branching orders within Boreotheria were fixed, as shown in Figure 1, because previous studies have resolved them unequivocally [12-16]. Additionally, we confirmed the validity of the phylogenetic relationships within Boreotheria using our dataset (see Additional data file 1 [Supplementary Text and Table S1]).

### Incongruent maximum likelihood tree provided by concatenate analyses

We mainly used the ML method because maximum parsimony and neighbor-joining analyses led to an apparently artificial tree with rodents at the basal position among eutherians, probably because of the long-branch attraction (see Additional data file 1 [Supplementary Text and Figure S1]). In contrast, the ML analyses supported the Boreotheria monophyly robustly. The concatenated dataset of the 2,789 gene sequences was analyzed at the nucleotide level with the GTR (General Time Reversible) + Γ_8 _and codon substitution [25] with Γ_4 _models, and at the amino acid level with the JTT-F (Jones-Tayor-Thornton (with the F-option)) + Γ_8 _model using the PAML version 3.15 [26] by fixing the relationships within Boreotheria, as shown in Figure 1.

Interestingly, quite different results were generated depending on the method. Phylogenetic analysis of the concatenated nucleotide sequence, which is a commonly used method in mammalian phylogenetics, supported tree 3 (the Afrotheria/Xenarthra clade) with extremely high significance (Table 1). The other two hypotheses (basal Afrotheria and basal Xenarthra) were strongly rejected (0.0% bootstrap probability [BP], *P *< 0.001 by the conservative weighted test of Shimodaira and Hasegawa [wSH]) [27]. Even though three codon positions were separately analyzed, each position consistently supported tree 3 as far as different genes were concatenated (Additional data file 1 [Table S2]). If we had concluded our analysis with these conventional methods, then tree 3 would have appeared to reflect an apparently true evolutionary history. With the codon substitution model, however, tree 3 was rejected (0.6% BP, *P *= 0.026 wSH) and tree 1 was the ML tree instead. By amino acid analysis, tree 2 was rejected (0.2% BP), and the other two hypotheses were nearly equally likely. Thus, our large concatenated dataset, comprising 2,789 genes (about 1 Mb), was very sensitive to the assumed model in rooting the eutherian tree.

**Table 1:** Comparison of the log-likelihood for the three hypotheses with each model

Concatenate or separate model	Substitution model	Tree	< ln *L *> (Δ ln *L *± *SE*)	KH	wSH	BP	#p	AIC
Concatenate model	GTR + Γ_8_	1	-117.2 ± 31.1	0.000	0.000	0.0		
		2	-147.3 ± 29.7	0.000	0.000	0.0		
		3	< -4,076,316.3 >			100.0	26	8,152,684.6
	Codon + Γ_4_	1	< -3,828,351.7 >			88.1	81	7,656,865.4
		2	-77.8 ± 64.5	0.112	0.185	11.3		
		3	-142.7 ± 65.0	0.014	0.026	0.6		
	JTT-F + Γ_8_	1	< -1,905,933.9 >			51.6	37	3,811,941.8
		2	-84.1 ± 37.4	0.014	0.028	0.2		
		3	-1.7 ± 41.9	0.478	0.637	48.2		

Separate model (among 2,789 genes)	GTR + Γ_8_	1	< -3,963,489.9 >			86.2	72,514	8,072,007.8
		2	-117.4 ± 72.3	0.050	0.092	4.1		
		3	-91.4 ± 72.7	0.104	0.174	9.7		
	Codon + Γ_4_	1	< -3,621,322.1 >			89.6	225,909	7,694,462.2
		2	-128.0 ± 103.2	0.107	0.164	10.4		
		3	-527.9 ± 96.3	0.000	0.000	0.0		
	JTT-F + Γ_8_	1	< -1,799,245.4 >			93.4	103,193	3,804,876.8
		2	-134.9 ± 88.5	0.064	0.112	6.6		
		3	-317.6 ± 85.5	0	0.000	0.0		

Maximum likelihood (ML) trees varied depending on the substitution model used for the concatenate analysis, whereas the separate model analyses consistently supported tree 1. The log-likelihood of the ML tree is given in angled brackets, and the differences in the log-likelihoods of alternative trees from that of the ML tree ± 1 standard error were estimated using the formula of Kishino and Hasegawa [28]. Numbering of the trees corresponds to that shown in Figure 1. KH and wSH denote *P *values derived using by the test of Kishino and Hasegawa [28] and the weighted test of Shimodaira and Hasegawa [27], respectively, calculated by the CONSEL program [47]. AIC, the Akaike Information Criterion [29]; #p, number of parameters of the model.

### ML analysis using the separate method

Because our dataset was composed of a large number of genes, variations in the tempos and modes of evolution among genes were expected to be very large. Therefore, we next carried out ML analyses with the separate model, which takes account of this variety by assigning different parameters to different genes [28]. Interestingly, the nucleotide, amino acid, and codon substitution models all consistently supported tree 1 (Table 1). The separate model was superior to the concatenate model based on the Akaike Information Criterion (AIC) [29], except for the codon substitution model, in which separation into 2,789 genes might have introduced too many parameters.

We next categorized the 2,789 genes into several groups (5, 10, 56, 100, 200, 558, 930, 1,395, or 2,789 categories) according to their evolutionary rates, and performed the separate analyses, in which different parameters were assigned to each category. For this categorization, we assessed the evolutionary rate for each of the 2,789 genes from the total branch length (TBL) estimated by the ML analysis of the gene. Because the AIC tends to favor complex model (with high number of parameters), we also applied the second order correction of AIC (AICc) in this study. The AICc is recommended when the number of characters or sites (#s) is small compared to that of parameters (#p; in the case #s/#p < 40) [30,31]. We compared the log-likelihood and AIC (or AICc) among the results to find better model for the dataset (Table 2). At nucleotide level, separation into each of the 2,789 genes exhibited the smallest AICc, supporting tree 1 with a BP of 86% (basal-Afrotheria hypothesis). In the codon substitution model, separation into 100 categories supported tree 1 (BP = 94%) with the smallest AIC. At amino acid level, tree 1 was the ML tree with separation into 56 categories, although the support for tree 3 is comparable to that for Tree 1. Accordingly, all of the separate analyses among gene categories with the smallest AIC or AICc favored tree 1 (see bold type in Table 2).

**Table 2:** Comparison of BPs among trees 1 to 3 analyzed with concatenate and separate models

Model	#c	Ln L	#p	#s	#s/#p	AIC	AICc	Tree 1	Tree 2	Tree 3
Nucleotide (GTR + Γ_8_)	1	-4,076,316.3	26	1,011,870	38,918.1	*8,152,684.6*	8,152,684.6	0.0	0.0	100.0
	5	-4,059,904.9	130	1,011,870	7,783.6	*8,120,069.8*	8,120,069.8	0.0	0.0	100.0
	10	-4,058,547.6	260	1,011,870	3,891.8	*8,117,615.2*	8,117,615.3	0.0	0.0	100.0
	56	-4,055,469.5	1,456	1,011,870	695.0	*8,113,851.0*	8,113,855.2	0.1	0.0	99.9
	100	-4,053,634.1	2,600	1,011,870	389.2	*8,112,468.2*	8,112,481.6	0.1	0.0	99.9
	200	-4,049,237.9	5,200	1,011,870	194.6	*8,108,875.8*	8,108,929.5	0.2	0.0	99.8
	558	-4,035,535.0	14,508	1,011,870	69.7	*8,100,086.0*	8,100,508.1	1.7	0.0	98.3
	930	-4,022,303.0	24,180	1,011,870	41.8	*8,092,966.0*	8,094,150.0	3.6	0.0	96.4
	1,395	-4,006,623.4	36,270	1,011,870	27.9	8,085,786.8	*8,088,483.7*	25.0	0.7	74.3
	**2,789**	**-3,963,489.9**	**72,514**	**1,011,870**	**14.0**	**8,072,007.8**	***8,083,203.5***	**86.2**	**4.1**	**9.7**

Codon (+ Γ_4_)	1	-3,828,351.7	81	337,290	4,164.1	*7,656,865.4*	7,656,865.4	88.1	11.3	0.6
	5	-3,810,589.3	405	337,290	832.8	*7,621,988.6*	7,621,989.6	94.3	5.1	0.7
	10	-3,808,198.7	810	337,290	416.4	*7,618,017.4*	7,618,021.3	93.3	5.9	0.8
	56	-3,802,941.9	4,536	337,290	74.4	*7,614,955.8*	7,615,079.5	93.0	5.2	1.7
	**100**	**-3,799,324.6**	**8,100**	**337,290**	**41.6**	***7,614,849.2***	**7,615,247.9**	**94**.0	**4.9**	**1.1**
	200	-3,791,928.7	16,200	337,290	20.8	7,616,257.4	*7,617,892.2*	91.0	8.1	1.0
	558	-3,766,336.0	45,198	337,290	7.5	7,623,068.0	*7,637,056.1*	96.7	2.9	0.3
	930	-3,741,173.9	75,330	337,290	4.5	7,633,007.8	*7,676,332.8*	98.0	1.7	0.3
	1,395	-3,712,084.5	112,995	337,290	3.0	7,650,159.0	*7,764,009.4*	96.2	3.8	0.0
	2,789	-3,621,322.1	225,909	337,290	1.5	7,694,462.2	*8,610,876.3*	89.6	10.4	0.0

Amino acid (JTT-F + Γ_8_)	1	-1,905,933.9	37	337,290	9,115.9	*3,811,941.8*	3,811,941.8	51.6	0.2	48.2
	5	-1,879,320.4	185	337,290	1,823.2	*3,759,010.8*	3,759,011.0	63.4	0.2	36.5
	10	-1,877,405.7	370	337,290	911.6	*3,755,551.4*	3,755,552.2	63.9	0.3	35.9
	**56**	**-1,875,094.5**	**2,072**	**337,290**	**162.8**	***3,754,333.0***	**3,754,358.6**	**56.6**	**0.1**	**43.2**
	100	-1,873,607.4	3,700	337,290	91.2	*3,754,614.8*	3,754,696.9	58.7	0.5	40.9
	200	-1,870,213.5	7,400	337,290	45.6	*3,755,227.0*	3,755,559.0	59.8	0.2	40.1
	558	-1,858,842.6	20,646	337,290	16.3	3,758,977.2	*3,761,669.7*	81.2	1.1	17.7
	930	-1,847,528.8	34,410	337,290	9.8	3,763,877.6	*3,771,696.4*	81.6	6.5	11.9
	1,395	-1,834,624.0	51,615	337,290	6.5	3,772,478.0	*3,791,129.7*	87.1	10.9	2.0
	2,789	-1,799,245.4	103,193	337,290	3.3	3,804,876.8	*3,895,855.7*	93.4	6.6	0.0

Maximum likelihood (ML) analyses with nucleotide, codon, and amino acid substitution models and comparison of bootstrap probabilities (BPs) among trees 1 to 3. Concatenate (#c = 1) and separate analyses were performed for each dataset. The #c, #p, and #s represent the number of categories separated according to the total branch length of the 2,789 genes, the number of parameters, and the number of characters (or sites), respectively. AIC is the Akaike Information Criterion, and AICc is the AIC with second order correction. AIC with #s/#p > 40 and AICc with #s/#p < 40 are shown in italics. The best models based on AIC or AICc are shown in bold.

### Removal of fast-evolving gene data

**Figure 2:**
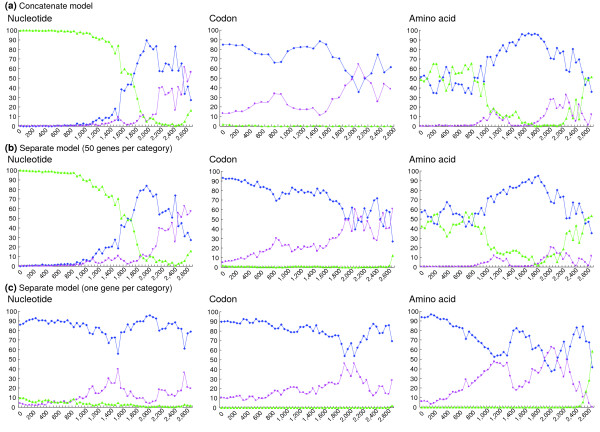
BPs of the three trees for the datasets constructed by successively removing the 50 most rapidly evolving genes. The horizontal axis shows the number of genes removed from the whole dataset of 2,789 genes. The dataset was analyzed using the **(a) **concatenate model; the **(b) **separate model, in which a category contains 50 genes grouped according to their total branch length; and **(c) **the separate model, in which different parameters were provided to each gene. Each analysis was performed using nucleotide (GTR + Γ_8_; the left-most column of panels), codon (+ Γ_4_; the middle column of panels), and amino acid (JTT + Γ_8_; the right-most column of panels) substitution models.

Because fast evolutionary rates are often associated with misleading effects, such as long-branch attraction [8,32], compositional bias, and heterotachy [3], we successively constructed datasets by removing the 50 most rapidly evolving genes at a time [8,32] (in terms of the TBL), finally producing 56 datasets. For each dataset, we first performed a concatenate analysis and monitored the shift in BP for each of the three trees. As expected, robust support (100% BP) for tree 3 showed a sharp decline to 0% BP by nucleotide analysis as the number of genes was reduced. In contrast, BPs for both trees 1 and 2, but particularly tree 1, increased (Figure 2a). In addition, the ambiguous support for trees 1 and 3 by the amino acid analysis shifted to reject tree 3 and stably support tree 1 (Figure 2c). Only for the concatenate analysis at codon level, we removed 100 genes at a time to produce 28 datasets (Figure 2b), and tree 3 was not supported with any dataset. These support levels became ambiguous when the majority of the genes were removed (> 2,600), but this was probably due to the extremely small number of remaining phylogenetically informative sites included in the slowly evolving genes.

Additionally, for each of the 56 datasets, we used the separate method so that a category includes 50 genes, and monitored the BPs as well. The shift of BPs for each tree was very similar to those of concatenate analysis with any model (Figure 2d-f). In the amino acid analysis, the separate analysis for this categorization (50 genes per category), using all of the 2,789 genes, showed ambiguous support for tree 1 and 3 with the smallest AIC, but removal of rapidly evolving genes was associated with decline in support for tree 3 (Figure 2f).

Furthermore, we conducted the separate analysis with separation into each gene along with the nucleotide, amino acid, and codon substitution models for each of the 56 datasets (Figure 2g-i). Note that the separate analysis among each gene showed the smallest AICc in the nucleotide analysis (Table 2). In this analysis, tree 3 was not supported in any model.

Therefore, our large dataset exhibit serious incongruence among models; tree 3 is strongly supported (100% BP) by a conventional method with a concatenate model of nucleotide analysis, whereas the separate model among each gene with the smallest AICc supported tree 1. Overall, tree 1 (basal Afrotheria) appeared to be the most likely tree by comparing BPs (Figure 2 and Table 1), but the alternative hypotheses cannot be dismissed. Hallstrom and coworkers [33] recently analyzed a dataset of 2,840 genes (> 2 Mb) with the concatenate model to resolve the root of the eutherian tree, and concluded that the most likely tree supports the monophyly of Xenarthra and Afrotheria (tree 3 in the present study). Based on our results, however, we believe that further analysis of their dataset with the separate model is necessary to take heterogeneity among the genes into account.

### Possible cause of the misled tree

There are several factors that can lead to an incorrect tree, even with use of genome-scale data: nucleotide or amino acid compositional bias [1,5,9]; long-branch attraction caused by unequal evolutionary rates among lineages [2,7,8,34]; sparse taxon sampling [2,4,8]; and heterotachy (the shift of position specific evolutionary rates) [8,32,35-39]. If the long branch attraction artifact was operating, then large differences among the relevant branch lengths would have been seen in the tree. In the tree 3 analyzed with concatenate GTR + Γ_8 _model (Additional data file 1 [Figure S2]), large differences in branch lengths are observed only in the rodents (mouse/rat) and cow lineages, which are within densely sampled Boreotheria. Concerning the compositional bias, significant differences are remarkable also in rodents and cow among eutherians (Additional data file 1 [Table S3]).

To examine whether the misled support for tree 3 resulted from the long branch attraction or compositional biases of the rodents and cow sequences, we performed a concatenate analysis with GTR + Γ_8 _model excluding the rodents (mouse and rat) and/or cow data. If the rodents and cow data provided such misleading effects as in our concatenate analysis shown in Table 1 and 2, then support for tree 3 should be reduced when we remove these sequences. Contrary to this expectation, however, tree 3 was still supported robustly (100% BP; Additional data file 1 [Table S4]). Therefore, we conclude that either the long branch attraction or the composition bias did not cause the misled support for tree 3. Furthermore, if they had actually caused the problem, it is not expected that the separate model could drastically improve the situation, as demonstrated in this work. We therefore expect that the heterogeneity among genes caused the problem.

If the inclusion of paralogous genes is causing the problem in our case, then it is expected that tree 3 supporting genes will tend to contain more paralogous comparisons, and accordingly their TBLs tend to be longer than average. We therefore investigated the distribution of TBLs of 848 genes that prefer tree 3, and compared the distribution with that of all 2,789 genes (Additional data file 1 [Figure S3]). The TBL was calculated using PAML 3.15 [26], with GTR + Γ_8 _model for each gene. However, no sign of more paralogs in the tree 3 supporting genes than others was observed (Additional data file 1 [Figure S3]). Therefore, the specific cause of the misled support for tree 3 remains unclear.

The number of genes that can be used for phylogenetic analysis becomes large when genome-scale data are used. We showed here an extreme case in which an analysis of a large concatenated dataset of genes yields different results depending on the substitution model used. In our analysis, the differing results were not due to long branch attraction and compositional bias, but probably to large variation in tempos and modes of evolution among genes. This serious pitfall is more difficult to detect than long branch attraction or compositional bias. Furthermore, we demonstrated that this hidden but probably common problem can be overcome using the separate model. Therefore, given that increasing the sequence length certainly reduces sampling error and that large amounts of data are very powerful in phylogenetic analyses, it must be noted that a simple concatenated dataset carries with it the possibility of a seriously misleading artifact. To estimate a true phylogenetic relationship, it is necessary to give close attention to the data analysis and to improve the method by explicitly taking into account variation in tempo and mode of evolution among different genes.

### Root of the eutherian tree

Rooting the eutherian tree is important in order to clarify when and where early eutherians evolved in association with ancient large-scale continental drift. With the best available models (the separate and concatenated codon substitution + Γ models), although tree 1 was preferred, we could not completely exclude the alternative hypotheses. Given that even the genome-scale sequence analyses with the best available model could not provide a definitive conclusion, as demonstrated in this paper, it is important to increase the species sampling and the number of genes in the phylogenetic analyses of sequence data with improved models of molecular evolution. Recently, it was demonstrated that extensive phylogenetic analysis with increased taxon sampling tends to prefer the concatenate model over the separate one based on AICc in the case of plant phylogeny [40]. Therefore, because dozens of mammalian genome sequencing projects are currently in progress, it may be possible that increased sampling will allow the root of the eutherian tree to be resolved without application of the completely separate model (among 2,789 genes). It is also important to apply more extensive and multilateral analyses such as retrotransposon insertion analysis [15,16,22,41] in order to maximize the explosively developing genomic data. In the near future, evolutionary history of mammals and its association with ancient continental drift will be resolved.

## Conclusion

The availability of large genomic sequence datasets for various mammals allows us to perform an extensive ML analysis of the phylogenetic relationship among Boreotheria, Xenarthra, and Afrotheria, in order to determine the root of eutherian tree based on 2,789 genes collected from ten mammalian species. Although a conventional method of concatenate analysis with a GTR + Γ model suggests the monophyly of Afrotheria and Xenarthra with 100% BP, this tree is rejected by ML analyses with the separate model, which takes into account the different tempos and modes of evolution among genes. We demonstrate that the separate model should be used for phylogenetic inference in cases of large variation in evolutionary features among different genes, such as for genome-scale data.

## Materials and methods

### Collection of the gene dataset

A large sequence dataset was collected using the following five steps: extraction of all exon sequences of greater than 200 bp from the human genome database; removal of duplicated (paralog) sequences from the human data; search of the armadillo and elephant genomic data for homologs of the human exons; collection of the homologous exons from other mammalian genomic data; and alignment of all of the sequences and removal of ambiguous nucleotide sites. Details for each step are shown below.

#### Step 1: extraction of all exon sequences of greater than 200 bp from the human genome database

We obtained human whole-genomic sequence data (version hg17) and an annotation data file (refFlat) for gene positions from the University of California, Santa Cruz Genome Bioinformatics database [42]. Protein-coding exon sequences of above 200 bp, identified from the annotation file, were used because it is difficult to evaluate the homology of short exon sequences by BLAST search.

### Step 2: removal of duplicated (paralog) sequences from the human data

To find and remove duplicated sequence data from the human exon data, we performed a pair-wise homology search among the exon sequences using the local Basic Local Alignment Search Tool (BLAST) program [43]. In this step, an exon sequence was removed from the sequence collection if a similar sequence, excepting the exon itself, was detected by the search in the human sequence data. The criterion for the similarity was set at an E-value of 1 × 10^-11^. Thus, each of the resulting 50,527 exons was regarded as a single-copy sequence in the human genome.

### Step 3: search of the armadillo and elephant genomic data for homologs of the human exons

We obtained whole-genome shotgun sequences of the nine-banded armadillo (*Dasypus novemcinctus*) and the African elephant (*Loxodonta africana*) from the DNA Data Bank of Japan. We next performed a local BLAST search with a cut-off of 1 × 10^-11 ^to obtain homologs of the human single-copy exon sequences from the two species. To avoid comparing paralogous exons, we removed the exon information from the collection if multiple sequences were detected in either of the two genomic datasets. However, failure to detect duplicated sequences does not guarantee that only orthologous comparisons were made, both because whole-genome data were not always available and because one of the duplicated genes in a genome may have been lost during evolution. Next, the regions shared among human, armadillo, and elephant were extracted for each of the 7,068 exons obtained.

### Step 4: collection of the homologous exons from other mammalian genomic data

Whole-genome pair-wise alignment data of human versus various animals are available in the University of California, Santa Cruz Genome Bioinformatics database. The seven mammalian species used for our data collection were chimpanzee (*Pan troglodytes*; data ver. panTro1), rhesus macaque (*Macaca mulatta*; rheMac1), mouse (*Mus musculus*; mm7), rat (*Rattus norvegicus*; rn3), dog (*Canis familiaris*; canFam2), cow (*Bos Taurus*; bosTau1), and opossum (*Monodelphis domestica*; monDom1). The orthologs of the human exons were obtained from the seven species by referring to the alignment data, and ten sequences that included sequences from human, armadillo, and elephant were obtained for each exon. To exclude possible pseudogenes from the analysis, we removed from the dataset any exon for which any of the species contained a stop codon in the middle of the sequence. The remaining 4,782 exons were used for the subsequent alignment and analysis.

#### Step 5: alignment of all of the sequences and removal of ambiguous nucleotide sites

All of the exon sequences were concatenated for each species to avoid the technical difficulty of alignment. We aligned the sequences using the blastz [44] and multiz [45] programs. Phylogenetic information can be taken into account in the alignment program, and thus, with the exception of the three hypotheses shown in Figure 1, we fixed the relationships of the mammalian species analyzed as follows: ((((((human, chimpanzee), macaque), (mouse, rat)), (dog, cow)), armadillo, elephant), opossum). Next, we divided the concatenated sequences into each exon and removed codons in which insertions and deletions were found for any species. When multiple exons were parts of the same gene in our dataset, we concatenated the exons and used the resulting concatenation as one gene sequence, thereby obtaining 3,148 genes in total. Because very short sequences of homologous exons were detected in the BLAST search (step 3) for some genes, such sequences (< 120 bp) were removed in the phylogenetic analysis that followed. We finally collected a 2,789 gene dataset composed of 1,011,870 bp (337,290 codons) for each species. Therefore, these gene sequences were different from the actual gene sequences because of removal of exons and codons that were ambiguous in the alignment. Our dataset is suitable for phylogenetic analysis in terms of both quality (exclusion of missing/ambiguous alignment codons, paralogs, and pseudogenes) and the quantity (> 1 Mb per species).

### Phylogenetic analysis with the ML method

ML analyses were carried out using Phylogenetic Analysis by Maximum Likelihood (PAML) version 3.15 package [26] at the nucleotide and amino acid levels with both the concatenate and separate models. The data were analyzed as nucleotide sequences with the GTR + Γ_8 _model and the codon-substitution + Γ_4 _model, or as amino acid sequences with the JTT-F + Γ_8 _model. The rate parameters of the GTR model, parameters of the codon substitution model, and the shape parameter (α) of the Γ distribution were optimized. In the concatenate analyses, the concatenated sequences (1,011,870 bp from 2,789 genes) were regarded as homogeneous, whereas in the separate analyses the differences among the gene categories or among the 2,789 genes were taken into account by assigning different parameters (branch lengths and other parameters of the substitution model, such as the shape parameter of the Γ model) to different categories or to different genes.

We performed the analyses by separating the 2,789 genes into 5, 10, 56, 100, 200, 558, 930, 1395, or 2789 (each gene) categories according to TBL estimated from ML analyses for each gene. In the latter analyses, log-likelihood scores for respective genes were estimated with PAML and then the total log-likelihood of the whole dataset was calculated with TotalML program in the MOLPHY [46] package. The test of Kishino and Hasegawa [28] and the wSH [27] were performed using the CONSEL program [47]. BPs shown in Tables 1 and 2 and in Additional data file 1 (Table S4) were calculated using the resampling estimated log-likelihood method [48] with 10,000 replications. The AIC [29] and the AICc were applied to evaluate the fitting of the model to the data.

### Removal of rapidly evolving gene data

In our data, rapidly evolving genes might cause artificial effects more extensively than slowly evolving genes [8], and paralogous genes might still be included among seemingly 'rapidly evolving' genes. To evaluate the influence of such genes, we constructed datasets by successively removing the 50 more rapidly evolving genes starting from the 2,789 gene dataset, producing 56 concatenated datasets. In this procedure, the evolutionary rate of each gene was evaluated from the estimated total branch length of the ML tree. We applied both the concatenate model and the separate model to each of the 56 datasets. In the concatenate model, ML analyses with the nucleotide (GTR + Γ_8_), amino acid (JTT-F + Γ_8_), and codon (with Γ_4_) substitution models were performed, and changes in relative BPs among the three hypotheses were monitored, as shown in Figure 2. In the concatenate analysis with the codon substitution model, we analyzed 28 datasets produced by removing 100 fast-evolving genes at a time. Because the number of replications for the BP calculation is changed in the default setting of the PAML package [26] depending on the length of the sequence analyzed, 500 and 10,000 replications were applied when 2,450 or fewer genes were removed and more than 2,450 genes were removed, respectively. We also used the nucleotide (GTR + Γ_8_), amino acid (JTT-F + Γ_8_), and codon (with Γ_4_) substitution models in the separate model analysis, in which different parameters were provided to each category (a category includes 50 genes; Figure 2d-f) or each gene (Figure 2g-i), and the total evidence was evaluated with the TotalML program in the MOLPHY package [46]. BPs in the separate model were calculated using the resampling estimated log-likelihood method with 10,000 replications.

## Abbreviations

AIC, Akaike Information Criterion; AICc, second order correction of AIC; BLAST, Basic Local Alignment Search Tool; bp, base pair; BP, bootstrap probability; GTR, General Time Reversible; JTT-F, Jones-Tayor-Thornton (with the F-option); Mb, megabase; ML, maximum likelihood; TBL, total branch length; wSH, weighted test of Shimodaira and Hasegawa.

## Authors' contributions

HN, NO and MH designed the study and wrote the paper. HN collected the sequence data. HN and MH analyzed the data.

## Additional data files

The following additional data are available with the online version of this paper. Additional data file 1 includes additional explanatory text and several additional tables and figures.

## Supplementary Material

Additional data file 1 Provided are additional explanatory text and several additional tables and figures. Click here for file
