# Supplementary material for: Rooting the eutherian tree: the power and pitfalls of phylogenomics
Source: Genome Biol. 2007 Sep 21;8(9):R199. doi: 10.1186/gb-2007-8-9-r199 (PMC2375037; doi:10.1186/gb-2007-8-9-r199)
Supplement: Additional data file 1 — Provided are additional explanatory text and several additional tables and figures. [file gb-2007-8-9-r199-S1.pdf]

Supplementary Information

for

"Rooting the eutherian tree: the power and pitfalls of phylogenomics"

Hidenori Nishihara, Norihiro Okada, and Masami Hasegawa

Contents

Supplementary Text 2
Supplementary Table S1 3
Supplementary Table S2 5
Supplementary Table S3 6
Supplementary Table S4 7
Supplementary Figure S1 8
Supplementary Figure S2 10
Supplementary Figure S3 11

Supplementary Text

Validity of the relationships within Boreotheria demonstrated by ML analysis

We performed an ML analysis for 105 topologies of the mammals to confirm the validity of fixing the topology in Boreotheria as shown in Figure 1. In this analysis, we used the concatenated amino acid sequence dataset with the JTT-F + Γ_8 model and analysed the relationship among primates, rodents, laurasiatherians (dog and cow), armadillo, and elephant using opossum as an outgroup. As shown in Table S1, all topologies other than the three hypotheses of interest (Tree 1–3) were significantly rejected. Because we gave the topology of Boreotheria in advance when aligning the sequences (see Method), this result is to be expected; nevertheless the MP and NJ analyses yielded the polyphyly of Boreotheria with some models (see below and Fig. S1).

Maximum parsimony (MP) and neighbor-joining (NJ) analyses

We also analysed our concatenated nucleotide and amino acid datasets by the MP and NJ methods. For the MP analysis, the PAUP* ver. 4.0 package [49] was used and the MP tree was searched by the branch-and-bound algorithm. The MEGA ver. 3.0 package [50] was used for the NJ analysis with the Tamura-Nei model. BPs were calculated with 500 replications for both analyses. In both analyses, rodents (mouse and rat) were estimated to be the basal lineage among eutherians (Fig. S1) for both nucleotide and amino acid sequences. However, this is obviously a misled tree due to long-branch attraction [34] because the rodents lineage shows a longer branch than the others, and because this tree is inconsistent with the well-established phylogenetic position of rodents [12–16] (see also our result in Table S1).

Legend to Supplementary Figures

Figure S1

MP trees (A and B) and NJ trees (C and D) analyzed using our concatenated dataset. (A) the MP tree based on the nucleotide dataset, (B) the MP tree based on the amino acid dataset, (C) the NJ tree based on the nucleotide dataset with the Tamura-Nei model, and (D) the NJ tree based on the amino acid dataset with the Poisson model.

Figure S2

The ML trees based on the concatenated dataset. (A) GTR + Γ_8 model, (B) JTT-F + Γ_8 model, and (C) codon-substitution + Γ_4 model (see also Table 1).

Figure S3

The distribution of total branch length (TBL) of 848 genes which prefer Tree 3 compared with that of all 2,789 genes. The TBL was calculated by PAML 3.15 [26] with GTR + Γ_8 model for each gene.

Supplementary Table S1. ML analyses for 105 trees based on the concatenated amino acid dataset (JTT-F + Γ_8 model).

Tree	–Diff. ln L	± SE	BP(%)	Topology
1	<1905933.9>	ML	51.61	(((Human,Chimp),Rhesus),(Mouse,Rat),(((Opo,Lox),Das),(Dog,Cow)))
2	84.1	37.4	0.20	(((Human,Chimp),Rhesus),(Mouse,Rat),(((Opo,Das),Lox),(Dog,Cow)))
3	1.7	41.9	48.19	(((Human,Chimp),Rhesus),(Mouse,Rat),((Opo,(Das,Lox)),(Dog,Cow)))
4	1451.2	118.4	0.00	((((Human,Chimp),Rhesus),((Dog,Cow),(Das,Lox))),(Mouse,Rat),Opo)
5	953.3	84.5	0.00	(((Human,Chimp),Rhesus),(Mouse,Rat),((Opo,(Dog,Cow)),(Das,Lox)))
6	2106	125.7	0.00	(((((Human,Chimp),Rhesus),(Das,Lox)),(Dog,Cow)),(Mouse,Rat),Opo)
7	1456.8	91.7	0.00	(((Human,Chimp),Rhesus),((Mouse,Rat),(Dog,Cow)),(Opo,(Das,Lox)))
8	1944.1	124.0	0.00	(((((Human,Chimp),Rhesus),(Dog,Cow)),(Das,Lox)),(Mouse,Rat),Opo)
9	884.6	86.3	0.00	(((Human,Chimp),Rhesus),(Mouse,Rat),(Opo,((Dog,Cow),(Das,Lox))))
10	1490.4	91.0	0.00	((((Human,Chimp),Rhesus),(Dog,Cow)),(Mouse,Rat),(Opo,(Das,Lox)))
11	1840.9	113.7	0.00	(((Human,Chimp),Rhesus),((Mouse,Rat),((Dog,Cow),(Das,Lox))),Opo)
12	1495.2	81.1	0.00	((((Human,Chimp),Rhesus),(Dog,Cow)),(Mouse,Rat),((Opo,Lox),Das))
13	1578.8	89.4	0.00	((((Human,Chimp),Rhesus),(Dog,Cow)),(Mouse,Rat),((Opo,Das),Lox))
14	2471.2	118.4	0.00	((((Human,Chimp),Rhesus),(Dog,Cow)),((Mouse,Rat),(Das,Lox)),Opo)
15	1470.8	81.6	0.00	(((Human,Chimp),Rhesus),((Mouse,Rat),(Dog,Cow)),((Opo,Lox),Das))
16	1555.3	89.8	0.00	(((Human,Chimp),Rhesus),((Mouse,Rat),(Dog,Cow)),((Opo,Das),Lox))
17	1777.6	92.7	0.00	(((Human,Chimp),Rhesus),(Mouse,Rat),(((Opo,Lox),(Dog,Cow)),Das))
18	2547.1	119.7	0.00	((((Human,Chimp),Rhesus),(Das,Lox)),((Mouse,Rat),(Dog,Cow)),Opo)
19	2893.8	142.0	0.00	((((Human,Chimp),Rhesus),(((Dog,Cow),Lox),Das)),(Mouse,Rat),Opo)
20	2815.4	143.0	0.00	((((Human,Chimp),Rhesus),(((Dog,Cow),Das),Lox)),(Mouse,Rat),Opo)
21	1908.2	104.9	0.00	(((Human,Chimp),Rhesus),(Mouse,Rat),(((Opo,Das),(Dog,Cow)),Lox))
22	2576.8	119.4	0.00	((((Human,Chimp),Rhesus),(Das,Lox)),(Mouse,Rat),(Opo,(Dog,Cow)))
23	3119.4	139.4	0.00	((((((Human,Chimp),Rhesus),(Dog,Cow)),Lox),Das),(Mouse,Rat),Opo)
24	2452.2	122.4	0.00	(((Human,Chimp),Rhesus),(((Mouse,Rat),(Das,Lox)),(Dog,Cow)),Opo)
25	3057.7	139.5	0.00	((((((Human,Chimp),Rhesus),(Dog,Cow)),Das),Lox),(Mouse,Rat),Opo)
26	2557.6	120.1	0.00	(((Human,Chimp),Rhesus),((Mouse,Rat),(Das,Lox)),(Opo,(Dog,Cow)))
27	2369.9	121.4	0.00	(((Human,Chimp),Rhesus),(((Mouse,Rat),(Dog,Cow)),(Das,Lox)),Opo)
28	2237.5	111.0	0.00	(((Human,Chimp),Rhesus),(Mouse,Rat),((Opo,((Dog,Cow),Das)),Lox))
29	2305.9	109.5	0.00	(((Human,Chimp),Rhesus),(Mouse,Rat),((Opo,((Dog,Cow),Lox)),Das))
30	2279.3	110.0	0.00	(((Human,Chimp),Rhesus),(Mouse,Rat),(((Opo,(Dog,Cow)),Lox),Das))
31	2268.9	110.9	0.00	(((Human,Chimp),Rhesus),(Mouse,Rat),(((Opo,(Dog,Cow)),Das),Lox))
32	1945.0	103.9	0.00	(((Human,Chimp),Rhesus),(Mouse,Rat),((Opo,Das),((Dog,Cow),Lox)))
33	3632.6	150.9	0.00	((((((Human,Chimp),Rhesus),Das),Lox),(Dog,Cow)),(Mouse,Rat),Opo)
34	3601.8	151.6	0.00	((((((Human,Chimp),Rhesus),Lox),Das),(Dog,Cow)),(Mouse,Rat),Opo)
35	2230.4	111.5	0.00	(((Human,Chimp),Rhesus),(Mouse,Rat),(Opo,(((Dog,Cow),Lox),Das)))
36	1725.2	94.0	0.00	(((Human,Chimp),Rhesus),(Mouse,Rat),((Opo,Lox),((Dog,Cow),Das)))
37	2151.3	112.7	0.00	(((Human,Chimp),Rhesus),(Mouse,Rat),(Opo,(((Dog,Cow),Das),Lox)))
38	3421.3	136.1	0.00	(((((Human,Chimp),Rhesus),(Dog,Cow)),Das),((Mouse,Rat),Lox),Opo)
39	3453.4	128.7	0.00	(((Human,Chimp),Rhesus),((Mouse,Rat),((Dog,Cow),Das)),(Opo,Lox))
40	3486.9	151.2	0.00	(((((Human,Chimp),Rhesus),Lox),((Dog,Cow),Das)),(Mouse,Rat),Opo)
41	3533.9	134.9	0.00	(((((Human,Chimp),Rhesus),(Dog,Cow)),Lox),((Mouse,Rat),Das),Opo)
42	3583.7	149.7	0.00	(((((Human,Chimp),Rhesus),Das),((Dog,Cow),Lox)),(Mouse,Rat),Opo)
43	3587.5	149.5	0.00	(((((Human,Chimp),Rhesus),((Dog,Cow),Lox)),Das),(Mouse,Rat),Opo)
44	3603.8	151.7	0.00	((((((Human,Chimp),Rhesus),Lox),(Dog,Cow)),Das),(Mouse,Rat),Opo)
45	3552.5	150.8	0.00	((((((Human,Chimp),Rhesus),Das),(Dog,Cow)),Lox),(Mouse,Rat),Opo)
46	3149.9	134.2	0.00	((((Human,Chimp),Rhesus),(Dog,Cow)),((Mouse,Rat),Lox),(Opo,Das))
47	3422.0	150.4	0.00	(((((Human,Chimp),Rhesus),((Dog,Cow),Das)),Lox),(Mouse,Rat),Opo)
48	3273.7	138.0	0.00	(((Human,Chimp),Rhesus),((Mouse,Rat),(((Dog,Cow),Lox),Das)),Opo)
49	3110.8	124.5	0.00	((((Human,Chimp),Rhesus),(Dog,Cow)),((Mouse,Rat),Das),(Opo,Lox))
50	3722.8	136.2	0.00	(((Human,Chimp),Rhesus),((Mouse,Rat),((Dog,Cow),Lox)),(Opo,Das))
51	3198.1	139.0	0.00	(((Human,Chimp),Rhesus),((Mouse,Rat),(((Dog,Cow),Das),Lox)),Opo)
52	3555.3	135.7	0.00	((((Human,Chimp),Rhesus),(Dog,Cow)),(((Mouse,Rat),Das),Lox),Opo)
53	3466.4	137.3	0.00	((((Human,Chimp),Rhesus),(Dog,Cow)),(((Mouse,Rat),Lox),Das),Opo)
54	3319.0	132.3	0.00	(((((Human,Chimp),Rhesus),(Dog,Cow)),Lox),(Mouse,Rat),(Opo,Das))
55	3147.2	124.8	0.00	(((((Human,Chimp),Rhesus),(Dog,Cow)),Das),(Mouse,Rat),(Opo,Lox))

56	3474.5	128.7	0.00	(((Human,Chimp),Rhesus),(((Mouse,Rat),(Dog,Cow)),Das),(Opo,Lox))
57	3685.1	136.4	0.00	(((Human,Chimp),Rhesus),(((Mouse,Rat),(Dog,Cow)),Lox),(Opo,Das))
58	4010.0	145.8	0.00	((((Human,Chimp),Rhesus),Lox),((Mouse,Rat),((Dog,Cow),Das)),Opo)
59	4128.3	144.1	0.00	((((Human,Chimp),Rhesus),Das),((Mouse,Rat),((Dog,Cow),Lox)),Opo)
60	3519.2	130.7	0.00	(((Human,Chimp),Rhesus),(((Mouse,Rat),Das),(Dog,Cow)),(Opo,Lox))
61	3958.3	143.4	0.00	((((Human,Chimp),Rhesus),Lox),(((Mouse,Rat),(Dog,Cow)),Das),Opo)
62	3620.9	140.4	0.00	(((Human,Chimp),Rhesus),(((Mouse,Rat),Lox),(Dog,Cow)),(Opo,Das))
63	4019.4	142.2	0.00	((((Human,Chimp),Rhesus),Das),(((Mouse,Rat),(Dog,Cow)),Lox),Opo)
64	3903.1	148.1	0.00	(((((Human,Chimp),Rhesus),Das),(Dog,Cow)),((Mouse,Rat),Lox),Opo)
65	3768.6	147.5	0.00	((((Human,Chimp),Rhesus),((Dog,Cow),Das)),((Mouse,Rat),Lox),Opo)
66	3438.8	140.6	0.00	(((Human,Chimp),Rhesus),((Mouse,Rat),Lox),((Opo,Das),(Dog,Cow)))
67	3990.3	145.6	0.00	((((Human,Chimp),Rhesus),((Dog,Cow),Lox)),((Mouse,Rat),Das),Opo)
68	4015.3	148.0	0.00	(((((Human,Chimp),Rhesus),Lox),(Dog,Cow)),((Mouse,Rat),Das),Opo)
69	3367.6	128.8	0.00	(((Human,Chimp),Rhesus),((Mouse,Rat),Das),((Opo,Lox),(Dog,Cow)))
70	3448.1	128.1	0.00	((((Human,Chimp),Rhesus),Das),(Mouse,Rat),((Opo,Lox),(Dog,Cow)))
71	4049.1	147.4	0.00	((((Human,Chimp),Rhesus),Lox),(((Mouse,Rat),Das),(Dog,Cow)),Opo)
72	3974.4	143.9	0.00	(((((Human,Chimp),Rhesus),Lox),Das),((Mouse,Rat),(Dog,Cow)),Opo)
73	4001.6	143.1	0.00	(((((Human,Chimp),Rhesus),Das),Lox),((Mouse,Rat),(Dog,Cow)),Opo)
74	3605.7	138.8	0.00	((((Human,Chimp),Rhesus),Lox),(Mouse,Rat),((Opo,Das),(Dog,Cow)))
75	4005.3	145.6	0.00	((((Human,Chimp),Rhesus),Lox),(Mouse,Rat),((Opo,(Dog,Cow)),Das))
76	3905.7	146.4	0.00	(((Human,Chimp),Rhesus),(((Mouse,Rat),((Dog,Cow),Das)),Lox),Opo)
77	3551.4	138.3	0.00	((((Human,Chimp),Rhesus),Lox),((Mouse,Rat),(Dog,Cow)),(Opo,Das))
78	3684.0	149.3	0.00	(((Human,Chimp),Rhesus),(((Mouse,Rat),Lox),((Dog,Cow),Das)),Opo)
79	3994.2	143.9	0.00	((((Human,Chimp),Rhesus),Das),(Mouse,Rat),((Opo,(Dog,Cow)),Lox))
80	3514.7	131.2	0.00	(((((Human,Chimp),Rhesus),Das),(Dog,Cow)),(Mouse,Rat),(Opo,Lox))
81	3999.5	148.0	0.00	((((Human,Chimp),Rhesus),Das),(((Mouse,Rat),Lox),(Dog,Cow)),Opo)
82	3430.3	128.9	0.00	((((Human,Chimp),Rhesus),Das),((Mouse,Rat),(Dog,Cow)),(Opo,Lox))
83	4085.1	146.6	0.00	(((((Human,Chimp),Rhesus),Lox),Das),(Mouse,Rat),(Opo,(Dog,Cow)))
84	4112.2	145.9	0.00	(((((Human,Chimp),Rhesus),Das),Lox),(Mouse,Rat),(Opo,(Dog,Cow)))
85	3844.2	147.4	0.00	(((Human,Chimp),Rhesus),((Mouse,Rat),Lox),((Opo,(Dog,Cow)),Das))
86	3918.5	144.6	0.00	(((Human,Chimp),Rhesus),((Mouse,Rat),Das),((Opo,(Dog,Cow)),Lox))
87	4020.1	145.1	0.00	(((Human,Chimp),Rhesus),(((Mouse,Rat),((Dog,Cow),Lox)),Das),Opo)
88	3868.4	146.8	0.00	(((Human,Chimp),Rhesus),(((Mouse,Rat),Das),((Dog,Cow),Lox)),Opo)
89	3362.1	130.3	0.00	((((Human,Chimp),Rhesus),((Dog,Cow),Das)),(Mouse,Rat),(Opo,Lox))
90	3696.5	140.4	0.00	(((((Human,Chimp),Rhesus),Lox),(Dog,Cow)),(Mouse,Rat),(Opo,Das))
91	3651.9	137.7	0.00	((((Human,Chimp),Rhesus),((Dog,Cow),Lox)),(Mouse,Rat),(Opo,Das))
92	3794.8	147.5	0.00	(((Human,Chimp),Rhesus),((Mouse,Rat),Lox),(Opo,((Dog,Cow),Das)))
93	3856.1	144.0	0.00	(((Human,Chimp),Rhesus),((((Mouse,Rat),(Dog,Cow)),Das),Lox),Opo)
94	3962.8	145.6	0.00	((((Human,Chimp),Rhesus),Lox),(Mouse,Rat),(Opo,((Dog,Cow),Das)))
95	4005.4	147.6	0.00	((((Human,Chimp),Rhesus),Lox),((Mouse,Rat),Das),(Opo,(Dog,Cow)))
96	3916.6	143.2	0.00	(((Human,Chimp),Rhesus),((((Mouse,Rat),(Dog,Cow)),Lox),Das),Opo)
97	3935.3	144.2	0.00	(((Human,Chimp),Rhesus),((Mouse,Rat),Das),(Opo,((Dog,Cow),Lox)))
98	4013.5	143.4	0.00	((((Human,Chimp),Rhesus),Das),(Mouse,Rat),(Opo,((Dog,Cow),Lox)))
99	3924.5	148.4	0.00	((((Human,Chimp),Rhesus),Das),((Mouse,Rat),Lox),(Opo,(Dog,Cow)))
100	3754.4	147.7	0.00	(((Human,Chimp),Rhesus),((((Mouse,Rat),Das),Lox),(Dog,Cow)),Opo)
101	3947.9	147.8	0.00	(((Human,Chimp),Rhesus),((((Mouse,Rat),Das),(Dog,Cow)),Lox),Opo)
102	3659.5	149.1	0.00	(((Human,Chimp),Rhesus),((((Mouse,Rat),Lox),Das),(Dog,Cow)),Opo)
103	3893.6	148.8	0.00	(((Human,Chimp),Rhesus),((((Mouse,Rat),Lox),(Dog,Cow)),Das),Opo)
104	3877.5	146.2	0.00	(((Human,Chimp),Rhesus),(((Mouse,Rat),Das),Lox),(Opo,(Dog,Cow)))
105	3784.3	147.6	0.00	(((Human,Chimp),Rhesus),(((Mouse,Rat),Lox),Das),(Opo,(Dog,Cow)))

The log-likelihood of the ML tree is given in angled brackets, and the differences in the log-likelihoods of alternative trees from that of the ML tree ± SE were estimated using the formula of Kishino and Hasegawa [28]. Numbering of the trees corresponds to that shown in Figure 1. Mac, Arm, Ele and Opo in the table represent macaque, armadillo, elephant and opossum, respectively.

Supplementary Table S2. Separate analysis among three codon positions (GTR + Γ_8 model).

A. Comparison of the log-likelihood for the three phylogenetic hypotheses

Tree	< ln L > (Δ ln L ± SE)	KH	wSH	BP (%)	#p	AIC
1	–113.9 ± 33.6	0.000	0.000	0.1		
2	–131.3 ± 32.8	0.000	0.000	0.0		
3	<–3,919,511.0>			99.9	78	7,839,178.0

B. Log-likelihood for each position

Tree	1st	2nd	3rd	< ln L > (Δ ln L ± SE)
1	–22.7 ± 16.6	–28.5 ± 15.1	–62.7 ± 25.1	–113.9 ± 33.6
2	–33.1 ± 15.6	–44.2 ± 13.6	–54.0 ± 25.4	–131.3 ± 32.8
3	<–1,029,462.5>	<-850,474.9>	<-2,039,573.6>	<–3,919,511.0>

Supplementary Table S3. The χ^2 tests comparing the amino acid and nucleotide composition of each species to the average of the dataset.

A. Amino acid

Species	*P*-value of χ^2 test
Human	97.59%
Chimpanzee	97.35%
Macaque	97.56%
Mouse	0.00%
Rat	0.00%
Dog	59.92%
Cow	0.02%
Armadillo	91.13%
Elephant	99.99%
Opossum	0.00%

B. Nucleotide

(a) 1st codon positions

Species	*P*-value of χ^2 test
Human	39.93%
Chimpanzee	34.89%
Macaque	43.06%
Mouse	0.13%
Rat	0.03%
Dog	0.16%
Cow	0.00%
Armadillo	30.99%
Elephant	99.78%
Opossum	0.00%

(b) 2nd codon positions

Species	*P*-value of χ^2 test
Human	28.59%
Chimpanzee	29.27%
Macaque	23.16%
Mouse	0.11%
Rat	0.01%
Dog	29.54%
Cow	0.25%
Armadillo	92.38%
Elephant	82.46%
Opossum	0.00%

(c) 3rd codon positions

Species	*P*-value of χ^2 test
Human	0.00%
Chimpanzee	0.00%
Macaque	0.00%
Mouse	0.00%
Rat	0.00%
Dog	0.00%
Cow	0.00%
Armadillo	0.00%
Elephant	44.69%
Opossum	0.00%

TREE-PUZZLE program [51] was used.

Supplementary Table S4. Comparison of the log-likelihood for the three hypotheses with concatenate analysis (GTR+Γ_8) excluding cow and/or rodents (mouse/rat).

A. Excluding cow

Tree	< ln *L* > (Δ ln *L* ± *SE*)	KH	wSH	BP (%)
1	−147.6 ± 33.3	0.000	0.000	0.0
2	−178.6 ± 32.0	0.000	0.000	0.0
3	< −3,779,962.6 >			100.0

B. Excluding rodents (mouse/rat)

Tree	< ln *L* > (Δ ln *L* ± *SE*)	KH	wSH	BP (%)
1	−86.6 ± 28.0	0.001	0.002	0.1
2	−120.9 ± 26.3	0.000	0.000	0.0
3	< −3430106.9>			99.9

C. Excluding cow and rodents (mouse/rat)

Tree	< ln *L* > (Δ ln *L* ± *SE*)	KH	wSH	BP (%)
1	−125.2 ± 30.3	0.000	0.000	0.0
2	−159.8 ± 28.7	0.000	0.000	0.0
3	< −3127148.1>			100.0

A

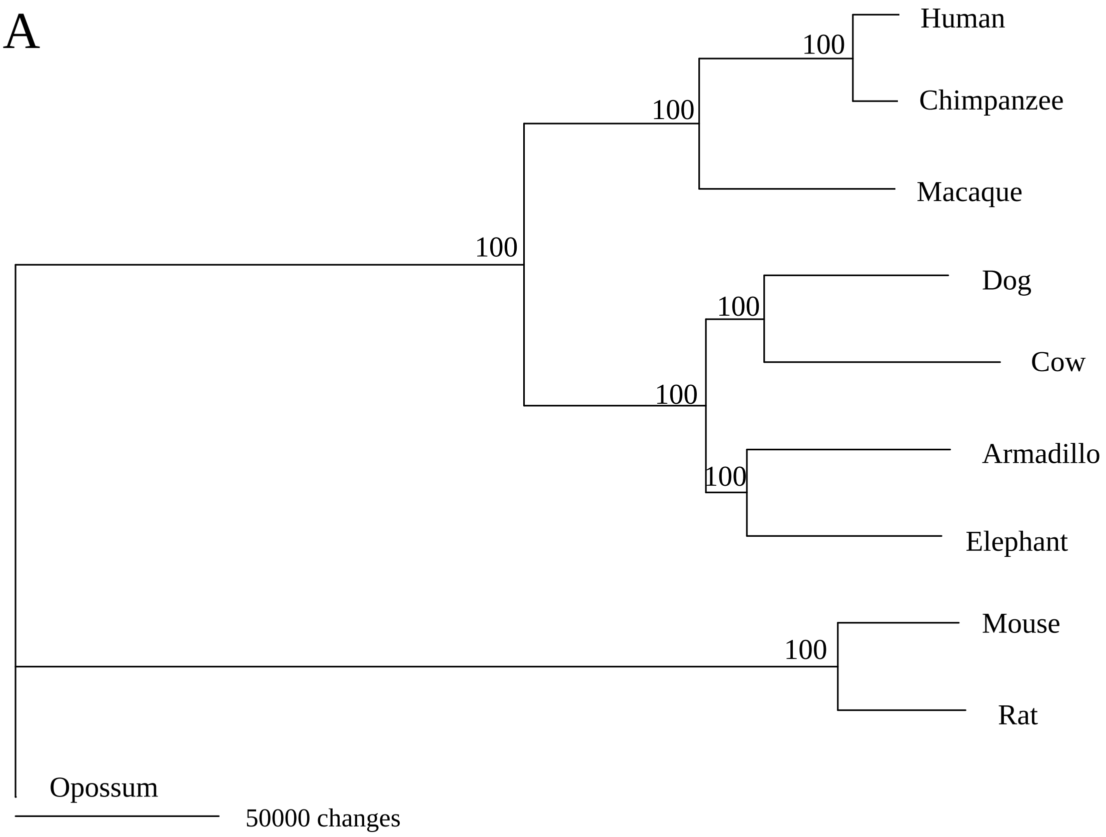
Human
Chimpanzee
Macaque
Dog
Cow
Armadillo
Elephant
Mouse
Rat
Opossum
100
100
100
100
100
100
100
50000 changes

B

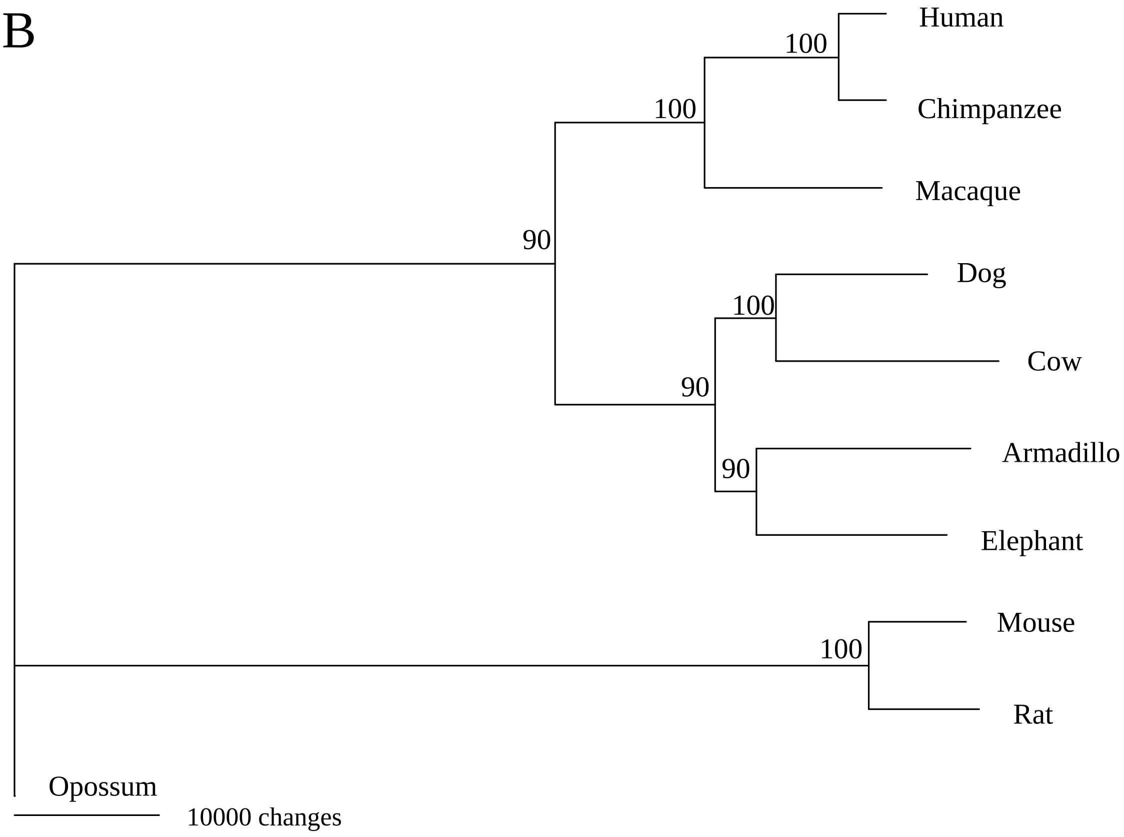
Human
Chimpanzee
Macaque
Dog
Cow
Armadillo
Elephant
Mouse
Rat
Opossum
100
100
90
100
90
90
100
10000 changes

C

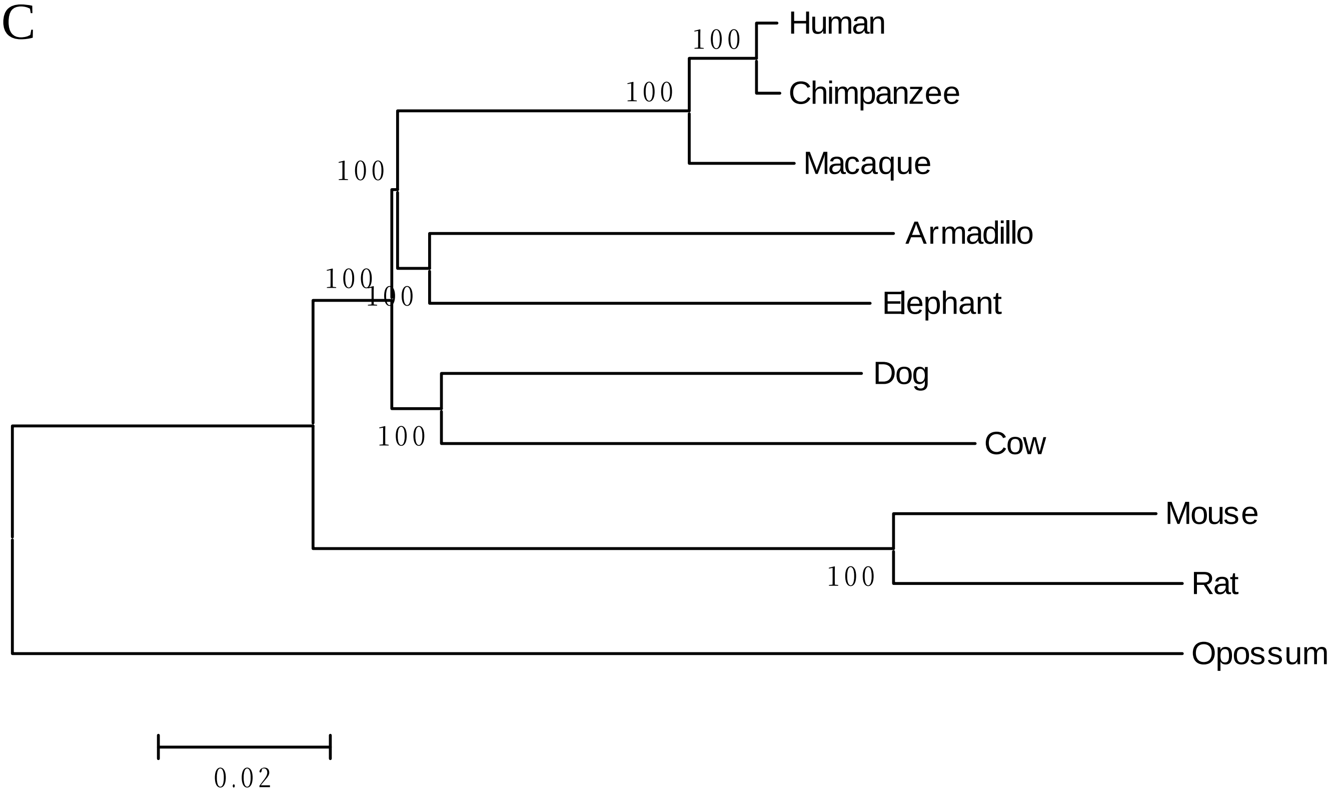
Human
Chimpanzee
Macaque
Armadillo
Elephant
Dog
Cow
Mouse
Rat
Opossum
100
100
100
100
100
100
100
0.02

D

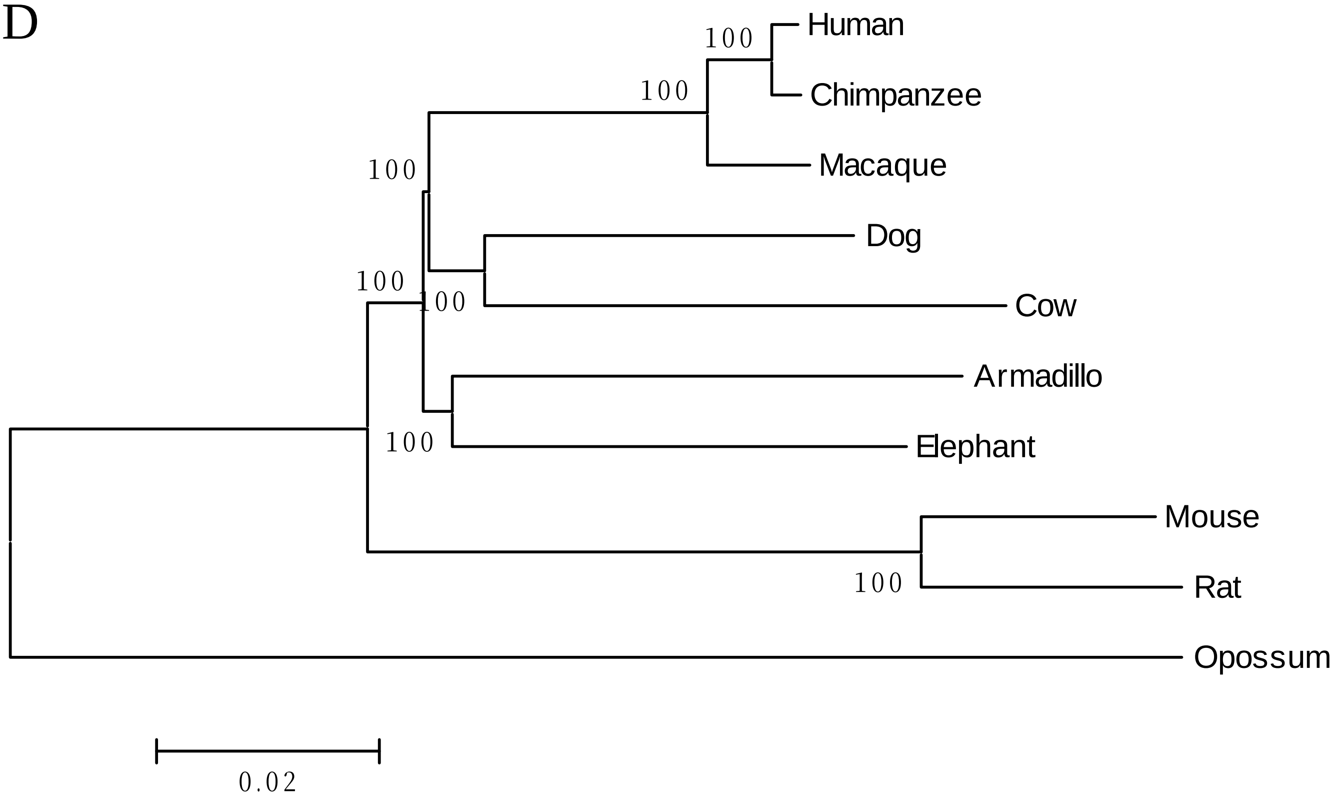
Human
Chimpanzee
Macaque
Dog
Cow
Armadillo
Elephant
Mouse
Rat
Opossum
100
100
100
100
100
100
100
0.02

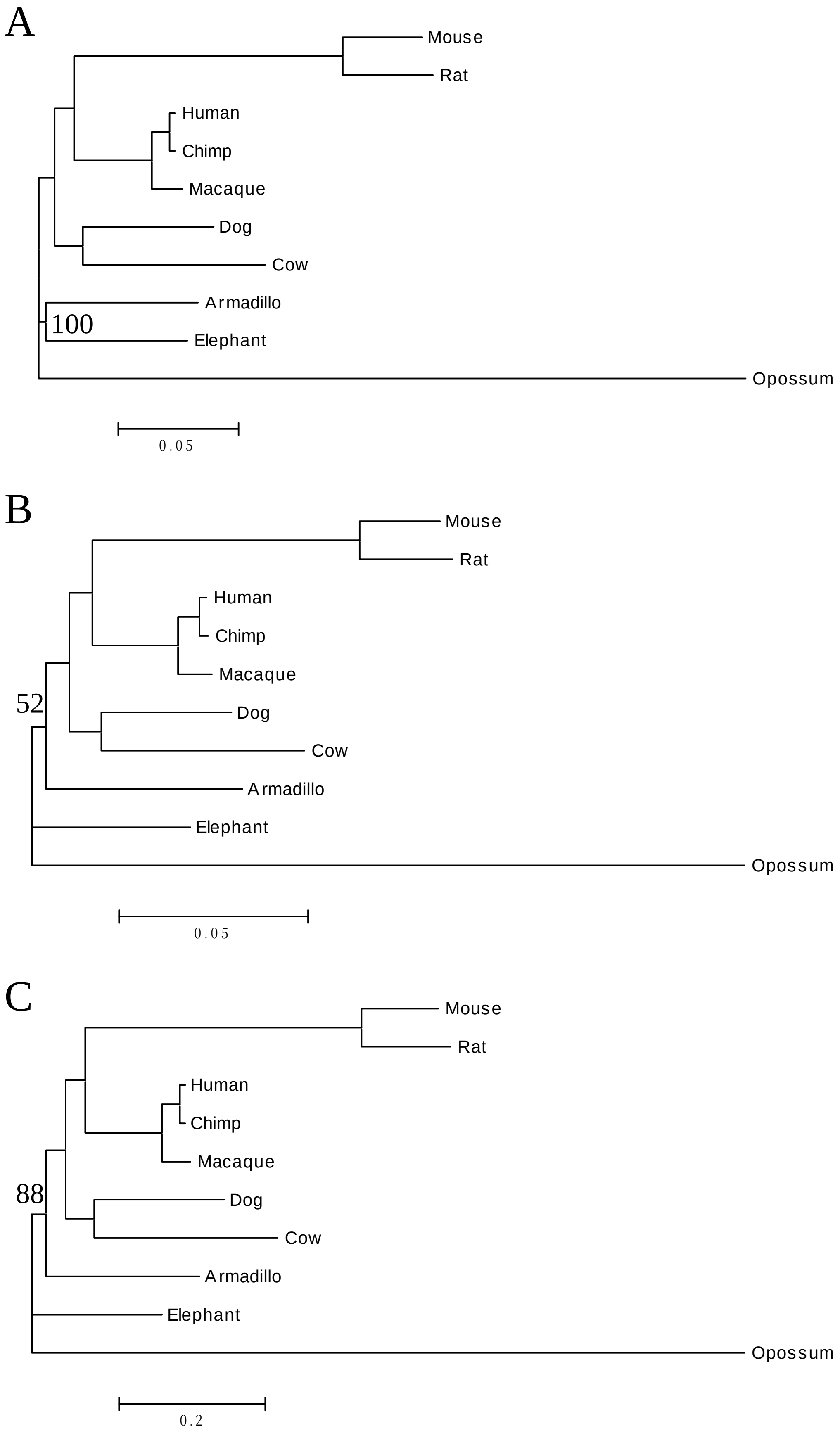
A
Mouse
Rat
Human
Chimp
Macaque
Dog
Cow
Armadillo
100
Elephant
Opossum
0.05
B
Mouse
Rat
Human
Chimp
Macaque
52
Dog
Cow
Armadillo
Elephant
Opossum
0.05
C
Mouse
Rat
Human
Chimp
Macaque
88
Dog
Cow
Armadillo
Elephant
Opossum
0.2

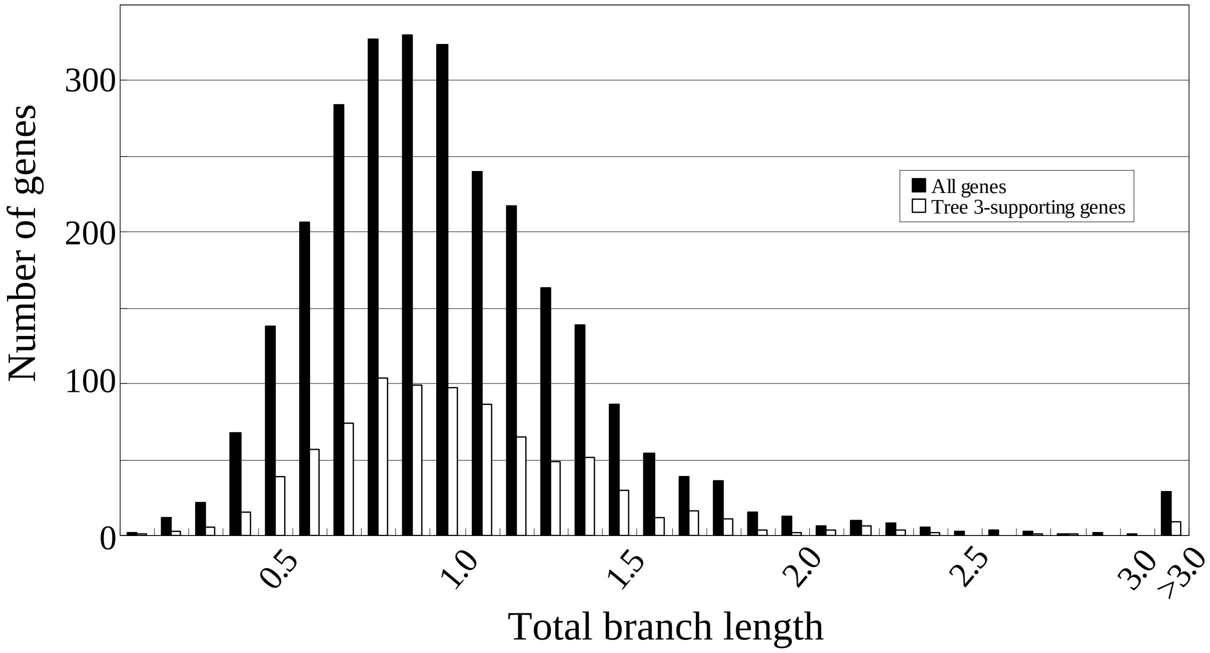

Number of genes
0
100
200
300
All genes
Tree 3-supporting genes
0.5
1.0
1.5
2.0
2.5
3.0
>3.0
Total branch length
